# Associations between teacher-student relationship and externalizing problem behaviors among Chinese rural adolescent

**DOI:** 10.3389/fpsyg.2023.1255596

**Published:** 2023-11-02

**Authors:** Shuping Yang, Xingchen Zhu, Wencan Li, Haohan Zhao

**Affiliations:** ^1^School of Education, Liaoning Normal University, Dalian, Liaoning, China; ^2^College of Psychology, Liaoning Normal University, Dalian, Liaoning, China; ^3^School of Chinese Language and Literature, Liaoning Normal University, Dalian, Liaoning, China

**Keywords:** teacher-student relationship, externalizing problem behaviors, peer relationship, mental health, parental knowledge, gender heterogeneity

## Abstract

The primary objective of this study is to present a fresh perspective on the correlation between teacher-student relationships and externalizing problem behaviors among adolescents. While previous research has examined this connection, there is still an insufficient understanding of the underlying mechanisms. Moreover, the crucial role of peer relationships, mental health, and parental knowledge has been overlooked. In this study, a total of 6,919 Chinese rural adolescents aged 13–19 years participated by completing an anonymous self-report questionnaire. The results show that: (1) teacher-student relationship has a protective effect against the development of externalizing problem behaviors; (2) peer relationship and mental health both have a mediating role in the relationship between teacher-student relationship and externalizing problem behaviors; (3) teacher-student relationship can indirectly affect externalizing problem behaviors through the chain mediation of peer relationship and mental health; (4) parental knowledge plays a moderating role between the teacher-student relationship and externalizing problem behaviors. As the level of parental knowledge increases among rural adolescents, the impact of the teacher-student relationship on externalizing problem behaviors becomes more pronounced; and (5) the impact of teacher-student relationship on externalizing problem behaviors has no significant gender differences. Given the study’s empirical outcomes, we discuss potential explanations and advocate for a comprehensive pedagogical approach to mitigate rural adolescent externalizing behaviors. This entails nurturing teacher-student relations, fostering inclusive peer environments, emphasizing mental health literacy, and synergizing with caregivers for a holistic home-school intervention.

## 1. Introduction

Problem behavior refers to atypical behavior exhibited by individuals that impedes their social adjustment (Achenbach et al., 1987). This encompasses both internalizing problem behaviors, such as anxiety and depression (Lee and Hankin, 2009), and externalizing problem behaviors, including truancy, fighting, alcohol abuse, and aggression (Petersen et al., 2015; Brock and Kochanska, 2016). Research indicates that the prevalence of externalizing problem behaviors, such as smoking and alcohol use, among Chinese adolescents in grades 7 to 12 ranges from 5.24 to 29.15% and has been increasing annually (Guo et al., 2019; Yuan and Wen, 2019; Chi and Cui, 2020). Numerous studies have demonstrated that externalizing problem behaviors can have detrimental short-term and long-term effects on adolescent health, functioning, and well-being. These effects include compromised brain function and structure (Lubman et al., 2015), academic difficulties (Park and Kim, 2015), mental health issues such as anxiety, depression, or addiction (Park and Kim, 2015), and even engagement in criminal behavior (Riala et al., 2004; Burt and Roisman, 2010). Self-determination theory posits that individuals are influenced by their social environment (Reeve et al., 2018), which aligns with social cognitive theory’s assertion that behavior is a result of the complex interplay between external environmental factors, internal personal characteristics, and past and present behavior (Bandura, 2001). Problem behavior theory (Jessor, 1987; Jessor et al., 1994) emphasizes that behavior is a product of human-environment interactions, particularly emphasizing the role of the external environment in prompting the expression of problem behaviors. The current investigation provides supporting evidence that the teacher-student relationship, as an exogenous environmental factor, can detrimentally impact various aspects of adolescents’ daily functioning, including work routines, academic achievements, familial connections, and emotional well-being (Lin and Tsai, 2002; Ryu et al., 2004; Ko et al., 2006; Leménager et al., 2018; Sha et al., 2019). Moreover, several studies consistently demonstrate a notable correlation between teacher-student relationship and the occurrence of behavioral issues (Beard and Wolf, 2001; Griffiths and Parke, 2002; Fontana et al., 2023).

According to the educational ecosystem theory, schools are the most important micro-environmental system for adolescents outside of their families (Bronfenbrenner, 1977; Bronfenbrenner and Morris, 2006). Teachers have a vital role in fostering the social development of adolescents by engaging with their students (Luckner and Pianta, 2011). Teachers, as important individuals for adolescents in school, are of great significance for their behavioral development (Hong and Eamon, 2012; Rudasill et al., 2018). Earlier studies have indicated that the caliber of students’ connections with their teachers is a fundamental aspect that affects the prosocial behavior of adolescents (Longobardi et al., 2021; Endedijk et al., 2022). Moreover, recent research has provided additional support to this perspective by demonstrating that establishing close relationships with teachers is positively correlated with reduced discipline problems, decreased aggressive behavior, and increased prosocial behavior even after a span of 4 years (Obsuth et al., 2017). On the other hand, consistent findings have shown that conflict within the student-teacher relationship is associated with various maladaptive outcomes, including heightened levels of aggressive behavior (Jungert et al., 2016) and increased instances of peer victimization (Lucas-Molina et al., 2015).

However, the specific effect of the teacher-student relationship on externalizing problem behaviors in adolescents remains understudied. Furthermore, insufficient scholarly attention has been given to comprehensively examining the underlying mechanisms through which teacher-student relationship affects externalizing problem behaviors among Chinese rural adolescents. Notably, the prevalence of problem behaviors is considerably higher among rural adolescents compared to their urban counterparts (Zhang et al., 2017; Wiggins et al., 2020; Zhang H. et al., 2021; Zhang Y. et al., 2021), indicating a significant disparity in problem behavior detection rates (Chen et al., 2022). Consequently, it becomes crucial to investigate and elucidate protective factors at both the environmental and individual levels to foster positive behavioral outcomes among Chinese rural adolescents. Furthermore, empirical research specifically focusing on rural adolescents from China is lacking. Thus, there is a pressing need to conduct a thorough investigation and elucidate the relationship between teacher-student relationship and externalizing problem behaviors among Chinese rural adolescents.

### 1.1. Peer relationship as a mediator

According to Santrock (1988), peers are individuals who share similar age, developmental stages, and maturity levels. Berk (1997) proposed a definition of peer relationships as connections between peers who possess equal status and levels of ability, enabling them to share common goals. Zhou et al. (2015) suggested that peer relationships are formed and developed through interactions between individuals of comparable psychological development. The ecosystem theory highlights the significance of the microsystem, such as the family and school, as external factors influencing individuals’ psychological development (Zhou et al., 2015). Among these microsystems, schools play a crucial role in adolescents’ lives, second only to families (Bronfenbrenner, 1992; Eccles and Roeser, 2011). Peer relationships, as a primary form of interpersonal connections within the school environment, exert a significant impact on adolescents’ development (Crosnoe, 2000; Giordano, 2003; Crosnoe and Johnson, 2011). This influence is particularly pronounced among Chinese adolescents, as schools serve as their primary learning and living environment, making interpersonal relationships with peers an influential factor that cannot be overlooked (Ren et al., 2011; Zhang et al., 2013).

The peer relationship plays a crucial role in adolescents’ socialization, reflecting both the level of intimacy and harmony with their peers, and serving as a significant indicator of their social adaptation (Qian et al., 2021). It holds immense importance for the development of adolescent behavior (Hartup, 1992). Peers possess the ability to identify problem behaviors sensitively, making peer relationships a significant influence on the occurrence of such behaviors (Fowler et al., 2015). Within peer groups, members tend to exhibit similarity in various adaptive issues, including externalizing and internalizing problem behaviors (Hogue and Steinberg, 1995; Espelage et al., 2003; Kiesner et al., 2003). Higher levels of peer acceptance motivate adolescents to learn prosocial behavior from their peers (Barry and Wentzel, 2006). Consequently, adolescents with high-quality peer relationships tend to display more prosocial behaviors (Carlo et al., 2012) and fewer problem behaviors (Bao et al., 2015). Conversely, poor peer relationships often lead to problem behaviors such as aggression and disciplinary violations (Palmqvist and Santavirta, 2006). Interventions targeting adolescents’ peer relationships can foster the development of their prosocial behavior (Yang et al., 2015).

Teachers’ influence on peer relationships has been likened to an “invisible hand” (Sakiz et al., 2012). Through supportive interactions, teachers have the ability to cultivate positive expectations of peer relationships among students and provide opportunities for adolescents to develop and practice social skills with their peers (Birch and Ladd, 1998). This teacher support contributes to the promotion of positive peer motivation in adolescents, including increased peer acceptance, likability, and a positive peer reputation (Van der Sande et al., 2018; Weyns et al., 2018). Importantly, the teacher-student relationship acts as a protective factor in adolescents’ developmental journeys, reducing the risk of aggressive behavior, bullying, and interpersonal harm (Cappella and Neal, 2012; De Laet et al., 2014). Through guidance, feedback, and reinforcement related to peer interactions, teachers effectively facilitate the development of prosocial behavior in adolescents (Patrick et al., 2012). Therefore, the teacher-student relationship to some extent influences peer relationships (Bierman, 2011; Ruzek et al., 2016).

Concerning the intricate dynamics involving teacher-student relationship, peer relationships, and externalizing problem behaviors, it is conceivable that peer relationships could potentially act as a mediating factor in the link between teacher-student relationship and externalizing problem behaviors.

### 1.2. Mental health as a mediator

Strong mental health and resilience in adolescents reduce the likelihood of problem behaviors occurring (Jones et al., 2013). Extensive research has established a common association between problem behaviors and poor mental health (Li et al., 2021). Wu’s (2000) study provides insights into the influence of mental health on problem behaviors in adolescents, highlighting how poor mental health serves as a significant trigger. Likewise, in a study conducted by Chen and Zhong (2012), it was discovered that insufficient mental well-being, characterized by factors such as stress, depression, or diminished self-esteem, significantly contributes to problem behaviors such as smoking, alcohol consumption, and engaging in physical altercations among adolescents.

Adolescence is the optimal period for psychological intervention (Ahmed et al., 2015). Based on educational ecosystem theory (Bronfenbrenner, 1977; Bronfenbrenner and Morris, 2006), the formation and development of mental health problems in junior high school students depend not only on their individual characteristics but also on the micro-environment they are directly exposed to. Schools are an important micro-environmental system that affects the development of adolescent students (Eccles and Roeser, 2011), and the impact of school environment on their mental health gradually increases (Jia and Liu, 2017). Teachers are one of the key factors in the school micro-system (Sakiz et al., 2012). Factors such as teaching methods, teacher’s teaching level, teacher-student relationship, and teacher style have been found to exhibit significant correlations with adolescent mental health, as demonstrated by Hu (1994). They are believed to provide adolescent students with a sense of psychological safety, allowing them to explore the surrounding environment and buffer developmental risks by helping them establish relationships with peers, regulating emotions, and using positive coping strategies (Kennedy and Kennedy, 2004).

With regard to the interplay between teacher-student relationship, mental health, and externalizing problem behaviors, it is plausible that mental health could potentially serve as a mediating factor in the relationship between teacher-student relationship and externalizing problem behaviors.

### 1.3. The chain mediating role of peer relationship and mental health

Adolescent peer relationships play a significant role in shaping mental health outcomes during this critical developmental period (Brown and Larson, 2009). The quality of peer interactions has been consistently linked to various aspects of psychological well-being, highlighting the importance of understanding the impact of these relationships on adolescents’ mental health (Parker and Asher, 1993; Demir et al., 2007).

Based on the preceding discussion, it can be inferred that there is a relationship between teacher-student relationship and peer relationships, a relationship between peer relationships and mental health, and a further relationship between mental health and externalizing problem behaviors. Consequently, teacher-student relationship may indirectly influence the externalizing problem behaviors through a chain-mediated effect involving peer relationships and mental health.

### 1.4. Parental knowledge as a moderator

Parental knowledge, also referred to as parental behavioral control or parental monitoring, encompasses a range of interconnected parental behaviors aimed at nurturing and overseeing children’s activities, companions, friendships, and whereabouts (Dishion and McMahon, 1998). The influence of parental knowledge on the development of adolescent behavior has gained increasing attention in recent years (McAdams et al., 2014; Jiang et al., 2016; Zhang H. et al., 2021; Zhang Y. et al., 2021). The understanding is that an individual should not be viewed in isolation but rather within a broader social context. Educational ecosystem theory highlights the role of the family in fostering adolescent development (Bronfenbrenner, 1986). In fact, parental knowledge acts as a powerful protective factor that can mitigate the effects of risky environments and reduce the occurrence of problem behaviors in adolescents (Lahey et al., 2008; Chen et al., 2016; Jiang et al., 2016). Furthermore, several studies have shown that parental knowledge can alleviate adolescent externalizing problem behaviors (Jantzer et al., 2015; Chang and Qin, 2018; Zhang H. et al., 2021; Zhang Y. et al., 2021). Parental knowledge flourishes within close and trusting parent–child relationships, which may be the most effective approach to reducing problem behaviors in adolescents (Kerr et al., 1999; Stattin and Kerr, 2000; Crouter et al., 2005).

Parental knowledge can serve as a bridge between home and school, facilitating communication and cooperation between parents and teachers. Studies have shown that when parents have a high level of knowledge about their children’s academic and socioemotional functioning, they are better able to collaborate with teachers in identifying and addressing their children’s needs (Grolnick and Slowiaczek, 1994; Fan and Chen, 2001). This collaboration, in turn, strengthens the quality of teacher-student relationship, as teachers can provide tailored instruction and support that aligns with the child’s unique profile (Shumow et al., 1998; Pomerantz et al., 2007). When students perceive support from their teachers, they tend to develop increased trust and respect towards their educators, leading to a greater willingness to establish more intimate relationships with them (Roorda et al., 2011). In addition, when parents are informed about their children’s school experiences, they are more likely to engage in positive parenting practices that reinforce the value of education and provide a supportive home environment (Reynolds et al., 2004; Fatimaningrum, 2021).

Concerning the interplay of teacher-student relationship, parental knowledge, and externalizing problem behaviors, it is plausible that parental knowledge may exert a moderating influence on the relationship between teacher-student relationship and externalizing problem behaviors.

### 1.5. Gender heterogeneity

According to social role theory, gender role expectations and socialization processes may contribute to gender differences in adolescent adjustment (Eagly et al., 2000). For girls, gender role expectations lead them to prioritize interpersonal communication and place a greater emphasis on the protective effects of positive relationships, despite conflict having a greater disruptive impact for them (Davies and Lindsay, 2004). Girls typically internalize their emotions in response to external conflict to maintain longer-lasting relationships and a healthier quality of relationships (Simon and Furman, 2010). Additionally, girls are more likely to exhibit restraint in displaying problem behaviors to conform to cultural stereotypes and moral evaluations (Bennett and Sani, 2008). In contrast, boys are more likely to express themselves through external behaviors, whether in relationship formation or in response to conflict, and are therefore more prone to problem behaviors in those contexts (Kinsfogel and Grych, 2004). From a moral cognitive perspective during adolescence, boys are more likely than girls to believe that aggression is appropriate, and they maintain a higher level of aggression as a result (Pellegrini and Long, 2002). Moreover, boys’ aggressive behaviors are more socially acceptable than girls’, and when aggression-related cognition is activated, boys usually exhibit behaviors characterized by aggression and dominance (Poulin and Boivin, 2000). For boys, violence and bold rule-breaking behaviors are perceived as markers of masculinity (Archer, 2006). In addition, boys’ higher levels of testosterone, height, and muscle strength, compared to girls, make them more inclined to solve problems with physical force, leading them to exhibit more externalized problem behaviors (Maras et al., 2003). Therefore, studies have found that problem behaviors are more prevalent and severe among boys than girls (Storvoll and Wichstrøm, 2002).

In general, there are many differences in the social and emotional development of boys and girls (Weyns et al., 2018). Boys tend to have lower levels of school engagement than girls (Lam et al., 2012). Their relationships with teachers also differ, with boys receiving lower levels of average teacher support compared to girls (Hamre and Pianta, 2001). This may be due to the fact that girls have stronger empathy skills than boys (Yan and Su, 2018) and actively seek to establish more interpersonal relationships with teachers (Spilt et al., 2012), while boys tend to exhibit more autonomy and confidence, resulting in girls receiving more teacher support than boys (Eccles and Roeser, 2011). Additionally, gender role socialization perspectives suggest that intimate relationships may be more beneficial for girls, as such relationships align with the social connections that girls are expected to value (Ewing and Taylor, 2009).

Herein, the impact of teacher-student relationship on externalizing problem behaviors may has gender differences.

### 1.6. The present study

Given that adolescent externalizing problem behaviors are influenced by multiple domains, including school, family, and individual factors, addressing and preventing such behaviors is challenging from a single-domain perspective. The aforementioned findings suggest that teacher-student relationship is a key predictor of externalizing problem behaviors, with peer relationships and mental health potentially serving as intermediaries between teacher-student relationship and such behaviors. The association between teacher-student relationship and externalizing problem behaviors may be contingent on the level of parental knowledge, such that higher levels of parental knowledge may serve as a moderator of this relationship. Additionally, the impact of teacher-student relationship on externalizing problem behaviors may also differ based on gender. This study contributes to the development of effective intervention plans for rural adolescent externalizing problem behaviors, a topic that has received little attention in the literature. To our knowledge, no prior research has examined the combined effects of teacher-student relationship, peer relationships, mental health, and parental knowledge on externalizing problem behaviors among rural adolescents. Moreover, previous research has not examined the gender differences in the effects of teacher-student relationship on rural adolescent externalizing problem behaviors. Drawing from the self-determination theory, social cognitive theory, problem behavior theory and educational ecosystem theory, this study expands the existing literature on teacher-student relationship and externalizing problem behaviors.

**Figure 1:**
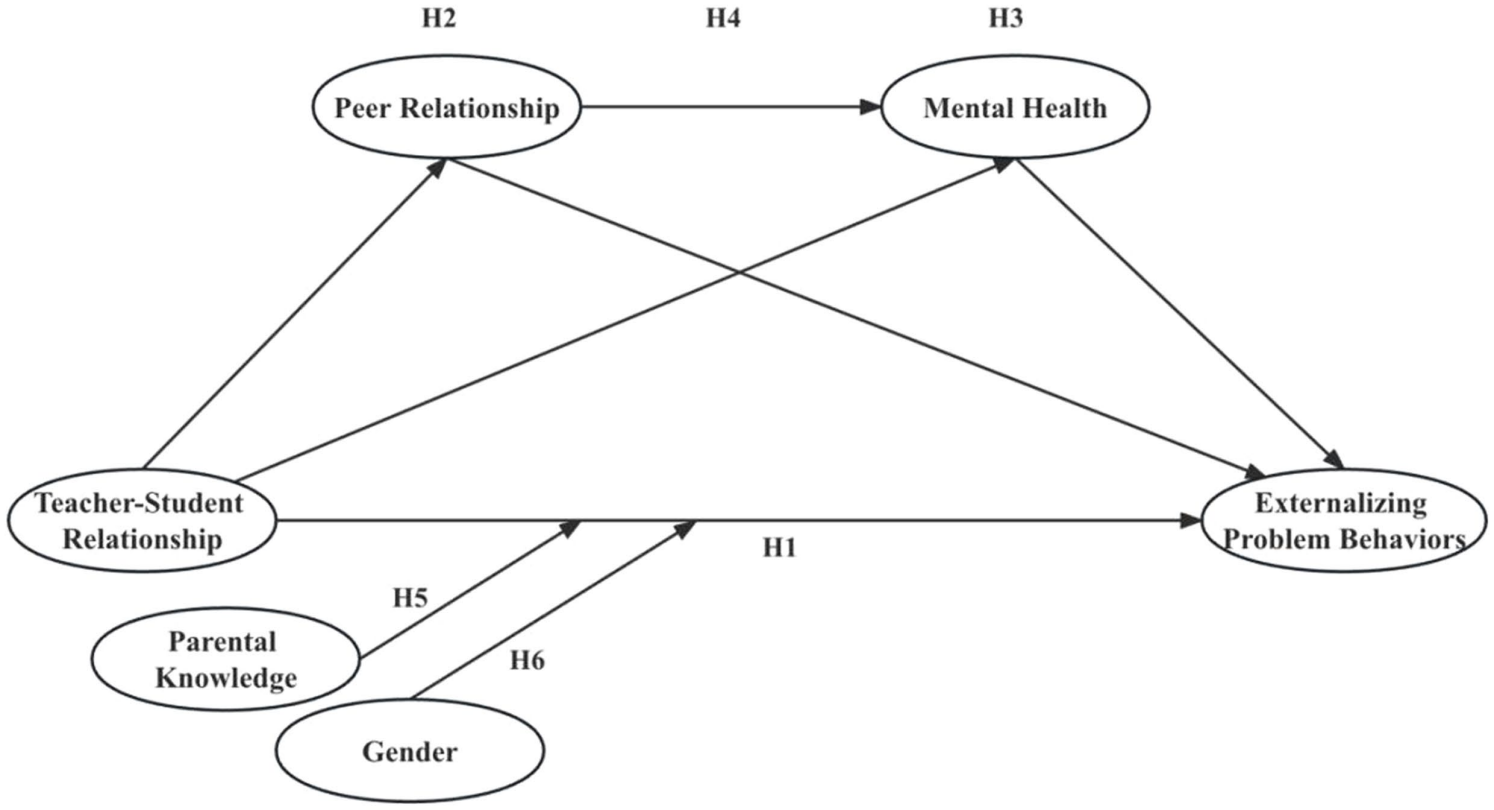
Research hypothesis model.

In line with our research goals, we put forward hypotheses ranging from *H1* to *H6*, as depicted in Figure 1.

*Hypothesis 1* (*H1*): Teacher-student relationship has a negative effect on the development of rural adolescent externalizing problem behaviors.

*Hypothesis 2* (*H2*): Peer relationship mediates the relationship between teacher-student relationship and rural adolescent externalizing problem behaviors.

*Hypothesis 3* (*H3*): Mental health mediates the relationship between teacher-student relationship and rural adolescent externalizing problem behaviors.

*Hypothesis 4* (*H4*): Peer relationship and mental health exhibit a chain mediating effect in the relationship between teacher-student relationship and rural adolescent externalizing problem behaviors.

*Hypothesis 5* (*H5*): Parental knowledge modifies the effect of teacher-student relationship on externalizing problem behaviors among rural adolescents.

*Hypothesis 6* (*H6*): The impact of teacher-student relationship on rural adolescent externalizing problem behaviors has gender differences.

## 2. Materials and methods

### 2.1. Participants

A total of 7,387 participants were recruited from eight counties in Liaoning, Henan, and Sichuan Province, China. These participants were selected from four junior and four senior high schools using stratified and random cluster sampling. Data collection occurred in late 2022. Permission was obtained from the schools, and both participants and their guardians provided written informed consent. The consent form did not require a signature or the student’s name, ensuring anonymity and confidentiality. Students were informed that their participation was voluntary, and they had the option to withdraw at any time. Trained research assistants supervised the students during school hours as they independently completed a pencil and paper questionnaire. The completed questionnaires were sealed in envelopes. A small gift, such as a pen or card, was provided as an incentive for participation. After a meticulous data curation process, 468 questionnaires were disqualified due to incomplete or absent responses. The final dataset, comprising 6,919 rigorously vetted questionnaires, yielded a commendable response rate of 94%. The participants’ ages ranged from 13 to 19 years (M = 14.514, SD = 0.687), with 49.6% (*N* = 3,431) being boys and 50.4% (*N* = 3,488) being girls. Out of the study cohort, 743 participants exhibited no manifestations of Externalizing problem behaviors. Consequently, the prevalence of Externalizing problem behavior within the sample stands at 89.3%. The university research ethics committee of the corresponding author reviewed and approved all research materials used in this study.

### 2.2. Measures

Externalizing problem behaviors. The measurement of externalizing problem behaviors was conducted using the Youth Self-Report (YSR; Achenbach, 1991a,b), which has been established as reliable and valid in previous studies (Patrick et al., 2011; Yang and Zhu, 2023). The YSR Externalizing Problem Behavior Scale comprises two subscales: aggressive behavior and rule-breaking behavior. Each subscale consists of 15 items, resulting in a total of 30 items. An example item is “I destroy my own things.” Participants rated each item on a 3-point Likert scale, with 1 indicating “not true,” 2 indicating “somewhat true,” and 3 indicating “very true.” Scores were obtained by calculating the average across all items, where higher scores indicated a higher level of externalizing problem behaviors among adolescents. The indices of confirmatory factor analysis (CFA) showed a good fit for the 2-factor model: CFI = 0.971, TLI = 0.937, RMSEA = 0.070, SRMR = 0.048. Furthermore, this measurement exhibited strong internal reliability, with a Cronbach’s alpha coefficient of 0.927.

Teacher-student relationship. The Teacher-Student Relationship Scale, originally developed by Pianta (1994) and later revised by Zou et al. (2007) for the Chinese context, is widely utilized in the assessment of teacher-student relationships in China. The revised questionnaire consists of 23 items, encompassing four dimensions: intimacy, conflict, support, and satisfaction. It is student-rated and has been extensively employed in recent studies on teacher-student relationships, such as those conducted by Wu and Zhang (2022) and Tan et al. (2022). Respondents use a 5-point Likert scale, ranging from 1 (completely disagree) to 5 (completely agree), with the conflict dimension reverse-scored before computing the average with other dimensions. Higher scores indicate a better teacher-student relationship. The indices of CFA showed a good fit for the 4-factor model: CFI = 0.955, TLI = 0.938, RMSEA = 0.068, SRMR = 0.053. The scale demonstrates good internal consistency, with a Cronbach’s alpha of 0.953.

Peer relationship. Peer relationship was measured using the 8-item PROMIS Pediatric Peer Relationships Scale (DeWalt et al., 2013), which demonstrated good psychometric characteristics for the Chinese adolescents (Huang et al., 2021). Example items are, “I was able to count on my friends” and “I felt accepted by other kids my age.” Items were rated on a 5-point Likert scale ranging from 1 (never) to 5 (almost always). Through calculating the average of all items, a higher score indicated a higher level of peer relationship. The indices of CFA showed a good fit for the 8 items: CFI = 0.985, TLI = 0.970, RMSEA = 0.058, SRMR = 0.025. And this measure showed good internal reliability, with a Cronbach’s alpha of 0.893.

Mental health. Mental health was measured using the 12-item General Health Questionnaire (GHQ-12) (McDowell, 2006), which has been widely used among Chinese adolescents (Ren and Zhu, 2022; Yang and Zhu, 2023). This measure comprises 12 items, equally divided into positive (e.g., “able to concentrate on whatever I do”) and negative (e.g., “insomnia due to anxiety”) domains. Response options on a 4-point Likert scale range from 1 (never) to 4 (often). The negative mental health items were reverse-scored prior to the calculation of a mean score across all items, where higher scores indicate better mental health. The indices of CFA showed a good fit for the 12 items: CFI = 0.978, TLI = 0.968, RMSEA = 0.063, SRMR = 0.046. And this measure showed good internal reliability, with a Cronbach’s alpha of 0.917.

Parental knowledge. Parental knowledge was assessed using a 5-item scale derived from the parental monitoring questionnaire developed by Stattin and Kerr (2000), which has been shown to possess good reliability and validity for Chinese adolescents (Jiang et al., 2016; Zhang H. et al., 2021; Zhang Y. et al., 2021). An illustrative item from the scale is “Do your parents know what activities you engage in during your free time?.” Participants rated each item on a 3-point Likert scale, ranging from 1 (know little) to 3 (know much). An overall score was obtained by calculating the average across all items, with higher scores indicating higher levels of parental knowledge. The indices of CFA showed a good fit for the 5 items: CFI = 0.989, TLI = 0.978, RMSEA = 0.054, SRMR = 0.017. Moreover, this measurement exhibited acceptable internal reliability, as indicated by a Cronbach’s alpha coefficient of 0.793.

Control variables. Control variables were selected based on previous studies (Zhang H. et al., 2021; Zhang Y. et al., 2021; Yang and Zhu, 2023). Two categories of control variables were chosen, namely family characteristics and individual characteristics related to adolescent externalizing problem behaviors (Table 1).

**Table 1:** Measurement instructions for variables.

Variables	Variable description
**Core variables**	
Externalizing problem behaviors	Continuous variable
Teacher-student relationship	Continuous variable
Peer relationship	Continuous variable
Mental health	Continuous variable
Parental knowledge	Continuous variable
**Control variables**	
Family’s book collection	1 = few, 2 = relatively few, 3 = average, 4 = relatively many, and 5 = many
Father’s level of education	1 = no education, 2 = primary school, 3 = junior high school, 4 = technical secondary school/technical school, 5 = vocational high school, 6 = ordinary high school, 7 = junior college, 8 = bachelor’s degree, and 9 = master’s degree and above
Mother’s level of education	1 = no education, 2 = primary school, 3 = junior high school, 4 = technical secondary school/technical school, 5 = vocational high school, 6 = ordinary high school, 7 = junior college, 8 = bachelor’s degree, and 9 = master’s degree and above
Parental expectations of education	1 = drop out of school now, 2 = junior high school, 3 = technical secondary school/technical school, 4 = vocational high school, 5 = ordinary high school, 6 = junior college, 7 = bachelor’s degree, 8 = master’s degree, and 9 = PhD
Family’s economic conditions	1 = very difficult, 2 = relatively difficult, 3 = medium, 4 = relatively rich, and 5 = best
Gender	0 = male; 1 = female
Nationality	0 = Ethnic minority; 1 = Han ethnicity
Age	Continuous variable (unit: year)
Physical health condition	1 = very poor, 2 = relatively poor, 3 = medium, 4 = relatively good, and 5 = best

### 2.3. Statistical analysis

SPSS Version 23.0, STATA Version 16.0 and MPLUS Version 8.3 were used for data analysis. First, we used Harman’s one-way method to test for the presence of common method bias. Second, descriptive statistics and bivariate correlations were used to assess the relationship among core variables. Third, when analyzing the impact of teacher-student relationship on adolescent externalizing problem behaviors, the method of gradually increasing influencing factors was used for the OLS regression results. Fourth, we employed structural equation modeling to examine the hypothesized chain mediation model. In this particular model, teacher-student relationship was regarded as a predicted variable, while the peer relationship and mental health were treated as mediating variables. The outcome variable in this case was the externalizing problem behaviors. Mediation was considered if the 95% CI of the indirect effect did not include zero. Bootstrapped confidence intervals (CIs) based on 5,000 bootstrapped samples were used to determine the significance of indirect effects. Fifth, moderated analyses were performed using the PROCESS 4.0 macro for SPSS with covariates. Finally, the samples were divided according to gender of the rural adolescent. The significance of the heterogeneity was examined by Fisher permutation test in our analysis.

## 3. Results

### 3.1. Common method bias test

Harman’s one-way method was used to test for the existence of common method bias (Zhou and Long, 2004). Exploratory factor analysis resulted in 22 factors with eigenvalues >1. The principal factor accounted for 24.449% of the aggregate variance, remaining below the 40% benchmark frequently cited as pivotal, indicating that common method bias is not apparent. Therefore, data analysis can proceed.

### 3.2. Correlation analysis of variables

Pearson correlation analysis was employed to examine the bivariate correlations among the key variables. The outcomes are presented in Table 2, which includes the mean values, standard deviations (SDs), and Pearson correlation coefficients for the core variables. The results revealed significant associations among all the core variables. Externalizing problem behaviors exhibited negative correlations with the teacher-student relationship (*r* = −0.224, *p* < 0.001), peer relationship (*r* = −0.260, *p* < 0.001), mental health (*r* = −0.402, *p* < 0.001), and parental knowledge (*r* = −0.288, *p* < 0.001). The teacher-student relationship displayed positive correlations with the peer relationship (*r* = 0.312, *p* < 0.001), mental health (*r* = 0.187, *p* < 0.001), and parental knowledge (*r* = 0.325, *p* < 0.001). Furthermore, the peer relationship exhibited positive associations with mental health (*r* = 0.255, *p* < 0.001) and parental knowledge (*r* = 0.433, *p* < 0.001). Lastly, mental health demonstrated a positive correlation with parental knowledge (*r* = 0.201, *p* < 0.001).

**Table 2:** Correlation analysis between key variables.

	Externalizing problem behaviors	Teacher-student relationship	Peer relationship	Mental health	Parental knowledge
Externalizing problem behaviors	–				
Teacher-student relationship	−0.224 ***	–			
Peer relationship	−0.260 ***	0.312 ***	–		
Mental health	−0.402 ***	0.187 ***	0.255 ***	–	
Parental knowledge	−0.288 ***	0.325 ***	0.433 ***	0.201 ***	–
Mean	1.451	3.868	3.860	2.652	2.347
SD	0.322	0.607	0.926	0.648	0.556

*p < 0.05, **p < 0.01, ***p < 0.001.

### 3.3. OLS regression results

The results in Table 3 show that the teacher-student relationship has a positive effect on preventing the development of externalizing problem behaviors. Model (1) only includes the independent variable of teacher-student relationship. Model (2) introduces family characteristic variables based on Model (1). Finally, Model (3) introduces adolescent individual characteristic variables based on Model (2). In Models (1) to (3) of Table 3, the estimated coefficients for the teacher-student relationship are −0.119 (*p* < 0.001), −0.088 (*p* < 0.001), and − 0.079 (*p* < 0.001) respectively. These coefficients indicate that the teacher-student relationship negatively predicts rural adolescent externalizing problem behaviors.

**Table 3:** OLS regression results for the effect of teacher-student relationship on rural adolescent externalizing problem behaviors.

Variables	(1) Externalizing problem behaviors	(2) Externalizing problem behaviors	(3) Externalizing problem behaviors
Teacher-student relationship	−0.119 *** (0.007)	−0.088 *** (0.007)	−0.079 *** (0.007)
Family’s book collection		−0.031 *** (0.004)	−0.025 *** (0.004)
Father’s educational level		−0.003 (0.003)	−0.004 (0.002)
Mother’s educational level		−0.007 * (0.003)	−0.006 * (0.003)
Parental educational expectations		−0.023 *** (0.003)	−0.021 *** (0.003)
Family economic conditions		−0.013 (0.007)	−0.004 (0.007)
Age			0.016 ** (0.006)
Gender			0.090 ** (0.007)
Nationality			−0.040 ** (0.014)
Physical health condition			−0.035 *** (0.004)
Sample size	6,919	6,919	6,919
*R*^2^	0.050	0.091	0.121

*p < 0.05, **p < 0.01, ***p < 0.001. The robust standard errors are in parentheses.

### 3.4. Mediation analysis

**Figure 2:**
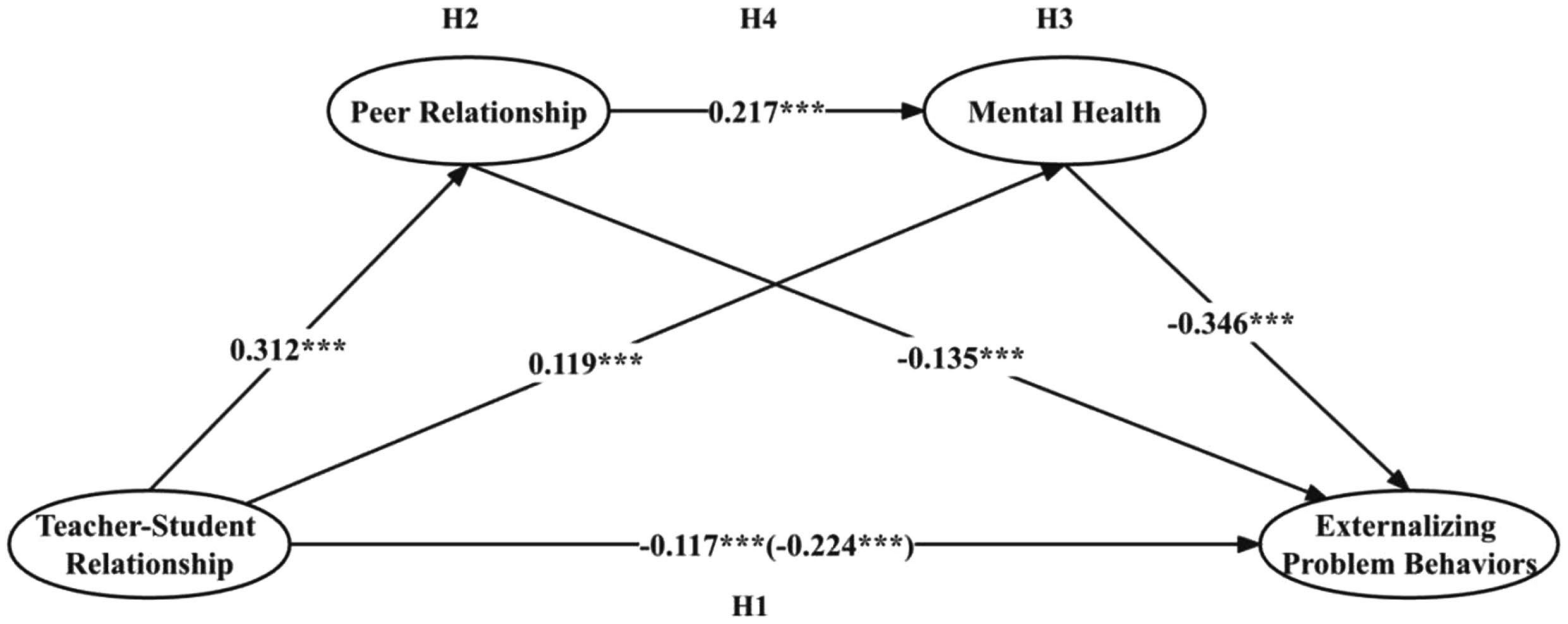
Results from path analysis on the research hypothesis model. Pathways between the variables are indicated by standardized beta estimates. *p <0.05, **p < 0.01, ***p < 0.001.

We calculated the mean values for the variables under scrutiny and subsequently constructed a structural equation model (SEM). The resulting Comparative Fit Index (CFI) and the Tucker-Lewis Index (TLI) both yielded values of 1, while the Root Mean Square Error of Approximation (RMSEA) and the Standardized Root Mean Square Residual (SRMR) returned values of zero. These metrics typically suggest that the research model employed in this study possesses some predictive validity. Therefore, a chain mediation model was tested, which consisted of three indirect effect as follows: (1) peer relationship played a mediating role in the relationship between teacher-student relationship and externalizing problem behaviors; (2) mental health mediated the relationship between teacher-student relationship and externalizing problem behaviors; and (3) teacher-student relationship can indirectly affect externalizing problem behaviors through the chain mediation of peer relationship and mental health (Figure 2).

The results showed teacher-student relationship and externalizing problem behaviors established significant and negative relationships (*β* = −0.224, *t* = −19.628, *p* < 0.001). After accounting for the influences of control variables, the direct impact of teacher-student relationship on externalizing problematic behavior remains significant and negative (*β* = −0.117, *t* = −9.589, *p* < 0.001). Teacher-student relationship was significantly and positively associated with peer relationship (*β* = 0.312, *t* = 26.226, *p* < 0.001), and peer relationship was significantly and negatively related to externalizing problem behaviors (*β* = −0.135, *t* = −10.280, *p* < 0.001). Teacher-student relationship was significantly and positively associated with mental health (*β* = 0.119, *t* = 9.049, *p* < 0.001), and mental health had a significant and negative influence on externalizing problem behaviors (*β* = −0.346, *t* = −28.220, *p* < 0.001). A significant and positive relationship between peer relationship and mental health was also identified (*β* = 0.217, *t* = 16.439, *p* < 0.001).

Furthermore, as seen in Table 4, the total effect of teacher-student relationship on externalizing problem behaviors was −0.224 (*SE* = 0.012, *95%* CI [−0.248, −0.200], *p* < 0.001) and the direct effect of −0.117 (*SE* = 0.012, *95%* CI [−0.142, −0.093], *p* < 0.001), showing that both the total effect and the direct effect were statistically significant. The indirect effect was −0.042 (*SE* = 0.004, *95%* CI [−0.051, −0.034], *p* < 0.001) in the pathway of teacher-student relationship → peer relationship → externalizing problem behaviors, and the mediation effect accounted for 18.834% of the total effect (−0.223). And the indirect effect was −0.041 (*SE* = 0.005, *95%* CI [−0.051, −0.032], *p* < 0.001) in the teacher-student relationship → mental health → externalizing problem behaviors, and the mediation effect accounted for 18.386% of the total effect. Finally, the indirect effect was −0.024(*SE* = 0.002, *95%* CI [−0.027, −0.020], *p* < 0.001) in the pathway of teacher-student relationship → peer relationship → mental health → externalizing problem behaviors, and the mediation effect accounted for 10.314% of the total effect. Given that the Bootstrap 95% confidence intervals do not encompass zero, the statistical significance of these three indirect effects is established. The data analysis revealed that the indirect impact of teacher-student relationship on externalizing problem behaviors was contingent upon peer relationship and mental health factors, which served as significant and positive partial mediators in the association between teacher-student relationship and externalizing problem behaviors.

**Table 4:** Direct, indirect, and total effects of the hypothesized model.

Model pathways	Estimated effect (*β*)	Boot SE	95% CI	
			Lower	Upper
**Direct effect**				
Teacher-student relationship → Externalizing problem behaviors	−0.117	0.012	−0.142	−0.093
**Indirect effects**				
Teacher-student relationship → Peer relationship → Externalizing problem behaviors	−0.042***	0.004	−0.051	−0.034
Teacher-student relationship → Mental health → Externalizing problem behaviors	−0.041***	0.005	−0.051	−0.032
Teacher-student relationship → Peer relationship → Mental health → Externalizing problem behaviors	−0.024***	0.002	−0.027	−0.020
Total effect	−0.224***	0.012	−0.248	−0.200

*p < 0.05, **p < 0.01, ***p < 0.001.

### 3.5. Moderated analysis

Table 5 presented findings indicating the moderating role of parental knowledge in the association between teacher-student relationship and externalizing problem behaviors. Firstly, the estimated coefficient for teacher-student relationship was −0.051 (*p* < 0.001), demonstrating statistical significance at the 1‰ level. Secondly, the estimated coefficient for the interaction term between teacher-student relationship and parental knowledge was −0.030 (*p* < 0.05).

**Table 5:** Moderated regression analyses predicating rural adolescent externalizing problem behaviors.

Variables	Externalizing problem behaviors
Teacher-student relationship	−0.051 *** (0.007)
Parental Knowledge	−0.125 *** (0.008)
Teacher-student relationship × Parental Knowledge	−0.030 * (0.012)
Control variables	Yes
Sample size	6,919
*R*^2^	0.161

*p < 0.05, **p < 0.01, ***p < 0.001. The robust standard errors are in parentheses.

**Figure 3:**
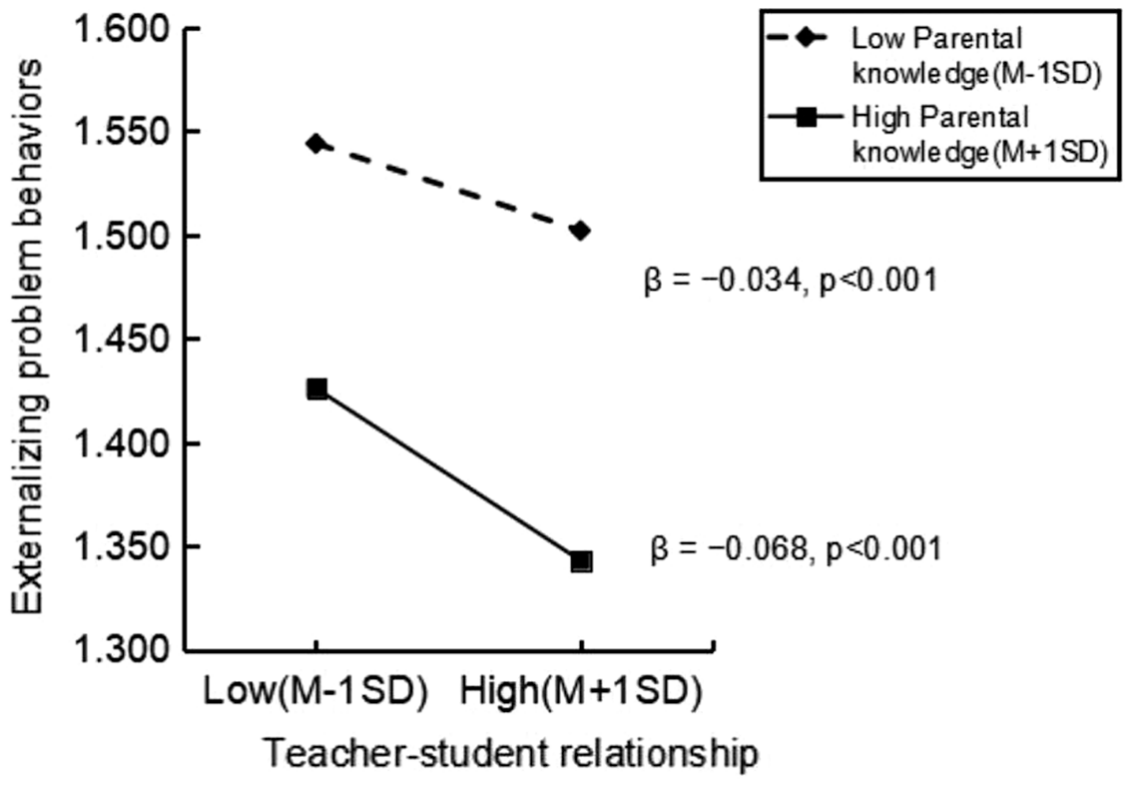
Simple slope analyses of the moderating effect of parental knowledge.

In accordance with the illustration presented in Figure 3, an in-depth investigation into the moderating aspect was conducted utilizing a simple slope analysis while establishing conditions of low (−1 SD) and high (+1 SD). The outcomes of this analysis substantiated the significance of the effect across all cohorts. Notably, when contrasting individuals with elevated parental knowledge against those with diminished parental knowledge, a stronger negative association between teacher-student relationship and externalizing problem behaviors was observed among the former group (*β* = −0.068, *p* < 0.001).

### 3.6. Heterogeneity results

The results in Table 6 showed there were no significant gender differences in the impact of teacher-student relationship on adolescent externalizing problem behaviors after controlling for control variables. Models (1) and (2) were the results of the impact of teacher-student relationship on the externalizing problem behaviors of male and female adolescents, respectively. The estimated coefficients passed the significance test of 1‰. The regression coefficient of the effect of teacher-student relationship on female adolescent externalizing problem behaviors was −0.081, which has a slightly higher absolute value than that of the effect of teacher-student relationship on male adolescent externalizing problem behaviors (−0.076). However, from the empirical *p* value obtained using the Fisher test on the gender coefficient difference in the impact of teacher-student relationship on adolescent externalizing problem behaviors did not pass the significance test. This demonstrated that the impact of teacher-student relationship on adolescent externalizing problem behaviors had no significant gender differences.

**Table 6:** Impact of teacher-student relationship on rural adolescent externalizing problem behaviors: gender-related differences.

Variables	(1) Externalizing problem behaviors	(2) Externalizing problem behaviors
	Male	Female
Teacher-student relationship	−0.076*** (0.011)	−0.081*** (0.008)
Control variable	yes	yes
Sample size	3,431	3,488
*R*^2^	0.092	0.118
Fisher empirical *p* value	0.388	

*p < 0.05, **p < 0.01, ***p < 0.001. The robust standard errors are in parentheses. The Fisher empirical *p* value is used to test the significance of the coefficient difference between the groups, which was obtained by 5,000 self-sampling times.

## 4. Discussion

### 4.1. Teacher-student relationship decreases the risk of externalizing problem behaviors in rural adolescents

The empirical evidence derived from this investigation provides affirmation for the initial hypothesis (H1) of the study, revealing that teacher-student relationship assumes a protective role in the deescalation of externalizing problem behaviors among rural adolescents. Invoking the foundational premises of attachment theory (Sroufe, 2005; Roorda et al., 2011), the texture and substance of the pedagogical dyad between educator and student emerge as critical determinants in the ontological trajectory of the learners. Within this relational nexus, imbued with nurturance and proximal closeness, students are afforded the scaffolding requisite to engender a sanguine, internalized working schema of self and others. This architectonics of relationality engenders not merely a transient sense of stability, but a durable ethos of emotional security, competency, and trust—traits that indelibly mark the individual’s broader social engagements. It is within this cocoon of affective assurance that adolescents are endowed with the moral and emotional capital to reciprocate nurturance and extend prosocial behaviors towards their peers and other constituents of their social microcosm. Furthermore, this relationship acquires heightened complexity as an anchoring locus, a “secure base” in the parlance of Bowlby’s attachment theory. Adolescents who thrive in this nurturing educational environment are prone to imbue their pedagogues with the status of a sanctuary, endowed with the capacity to furnish emotional ballast, promulgate affirmative behavioral templates, and catalyze the reinforcement of behaviors that harmonize with the ethical and academic objectives of the educational institution (Weinstein, 2002). Conversely, the pedagogical alliance’s dysregulation manifests in maladaptive sequela. Pedagogical relationships replete with conflict and affective disconnect precipitate not just transient affective upheavals but set into motion a cascade of adverse outcomes. Such schisms result in an unsettling sense of insecurity, emotional amplification typified by distress (Hamre and Pianta, 2001), and an attrition in the student’s reservoir of social competence (Pianta and Stuhlman, 2004; Rudasill et al., 2010). These resultant factors serve to potentiate the predilection for adolescents to manifest externalizing problem behaviors, thereby confirming an inversely proportional relationship between the quality of teacher-student relational bonds and the propensity for maladaptive adolescent behavioral manifestations.

### 4.2. The mediating effect of peer relationship and mental health between teacher-student relationship and externalizing problem behaviors in rural adolescents

This study also confirmed the second hypothesis (*H2*), which is the notion that peer relationship play a mediating role in understanding the association between teacher-student relationship and externalizing problem behaviors in rural adolescents. This finding underscores that a supportive and nurturing environment provided by teachers facilitates students’ ability to forge positive peer relationships. Importantly, these positive peer affiliations serve as a mediating mechanism, potentially acting as a protective buffer against the emergence of externalizing problem behaviors. Our conclusions resonate with the social development model’s tenets, emphasizing how positive peer associations play a pivotal role in curtailing problematic behaviors (Catalano et al., 2004). These outcomes also harmonize with the social support theory, suggesting that social affiliations significantly mitigate the adverse repercussions of stressors (Cohen and Wills, 1985). Within the purview of our investigation, it appears that positive peer interactions—stimulated by heightened teacher-student relationship quality—provide pivotal social support, attenuating the severity of externalizing problem behaviors among rural adolescents.

The third hypothesis (*H3*) of this study has been empirically validated, establishing mental health as the mediating mechanism through which teacher-student relationship influences the emergence of externalizing problem behaviors in rural adolescents. These findings imply that the effect of teacher-student relationship on externalizing problems is, at least partially, channeled through its impact on students’ mental health. Adolescents who receive higher levels of teacher-student relationship may experience better mental health outcomes, which, in turn, reduces the likelihood of engaging in externalizing behaviors. This mediating role of mental health can be explained by several mechanisms. First, teacher-student relationship has been shown to enhance students’ self-esteem and self-efficacy (Roorda et al., 2011; Hamre et al., 2013). Such positive self-perceptions are associated with improved mental health outcomes and lower levels of externalizing problems (Marsh et al., 2005; Barrett, 2013). Second, teacher-student relationship can foster a sense of belongingness and connectedness in students, which buffers against the development of mental health issues (Wentzel, 2009). Lastly, teacher-student relationship may indirectly promote mental health by mitigating the negative impact of stressors and adverse experiences, thereby reducing the likelihood of externalizing behaviors as maladaptive coping strategies (Eisenberg et al., 2009).

This study illustrates the chain mediating effect of peer relationship and mental health between teacher-student relationship and externalizing problem behaviors among rural adolescents (*H4*). In other words, adolescents with higher teacher-student relationship are more likely to develop externalizing problem behaviors through lower peer relationship and mental health. Peer relationships have been widely recognized as crucial factors influencing adolescent mental health outcomes (Buhrmester, 1990; Reijntjes et al., 2010). Positive peer relationships can provide social support, promote healthy emotional development, and facilitate adaptive coping strategies. Conversely, negative peer relationships, characterized by peer rejection, bullying, or social isolation, have been consistently associated with a range of mental health problems, including externalizing problem behaviors (Fergusson et al., 1996; Reijntjes et al., 2011). Therefore, the influence of peer relationships on mental health outcomes cannot be understated.

These findings serve as a practical and theoretical guide in promoting the cultivation of prosocial behavior among rural adolescents, emphasizing the crucial role played by peer relationship (Crosnoe and Johnson, 2011; Dishion and Tipsord, 2011; Ren et al., 2011; Zhang et al., 2013) and mental health (O’Gara et al., 2019; Li et al., 2021) in their overall well-being. Furthermore, our findings highlight the importance of considering peer relationships and mental health as a potential mechanism in interventions targeting teacher-student relationship and externalizing problem behaviors among rural adolescents. Interventions that aim to improve peer relationships, mental health and provide social support may effectively reduce the risk of externalizing problem behaviors associated with teacher-student relationship.

### 4.3. The moderating effect of parental knowledge between teacher-student relationship and externalizing problem behaviors in rural adolescents

When parents are more involved and knowledgeable about their children’s academic experiences, they may be more likely to encourage positive interactions with teachers and reinforce the value of academic success (Fan and Chen, 2001; Hill and Tyson, 2009). This can result in the teacher-student relationship being strengthened and more conducive to emotional support, which may have positive effects on adolescent behavior. In addition, parental knowledge may enhance the effectiveness of teacher-student relationship by providing teachers with more information about their students’ needs, interests, and social contexts (Pomerantz et al., 2007; Sheridan et al., 2013). Teachers who are more informed about their students’ lives may be better able to tailor their emotional support to meet their individual needs and provide guidance that is relevant and effective in reducing problem behaviors. This suggests that if parents have greater awareness of their children’s activities and social connections, teacher-student relationship may have a significant impact and ultimately result in reduced adolescent problem behaviors.

In accordance with the educational ecosystem theory (Bronfenbrenner, 1986), adolescents who benefit from high levels of positive family characteristics, such as parental knowledge, demonstrate a decreased likelihood of engaging in problem behaviors within high-risk environments, including teacher-student relationship. This phenomenon can be plausibly explained by considering that families characterized by elevated levels of parental knowledge foster an environment of harmony and warmth, wherein parents are more likely to provide effective guidance to their children, consequently resulting in lower levels of problem behaviors (Tian et al., 2019; Zhang H. et al., 2021; Zhang Y. et al., 2021). Consequently, it can be inferred that in cases where parents possess limited knowledge regarding their children’s activities and social relationships, teacher-student relationship can exact a significant toll, ultimately leading to heightened levels of externalizing problem behaviors.

### 4.4. The gender heterogeneity between teacher-student relationship and externalizing problem behaviors in rural adolescents

Teacher-student relationship has significant impact on the externalizing problem behaviors of male and female adolescents. However, there are no significant differences between genders, which rejects the sixth hypothesis (H6). A plausible explanation for the observed behavior patterns in adolescents is the fundamental similarity in relational needs across genders. Irrespective of being male or female, adolescents have inherent desires for connection, understanding, and belonging. The ways teachers nurture and establish relationships in educational settings can have profound implications for the overall well-being and academic performance of their students. Malecki and Demaray (2003) have illuminated the idea that teacher-student relationships can equivalently benefit both male and female students, contributing to a sense of security, engagement, and achievement motivation. However, while teacher-student relationships are pivotal, they are just one facet of the broader social environment impacting adolescent behaviors. Family environment emerges as another potent influence, possibly accounting for the gender differences observed in externalizing behavior problems among teenagers. The family, being the primary social unit, exerts significant influence over an adolescent’s development, behavior patterns, and coping mechanisms. Research by Deng et al. (2021), Moylan et al. (2010), Wang et al. (2021), and Chi and Cui (2020) underscores the multifaceted nature of familial influences, ranging from parenting styles, sibling dynamics, socio-economic conditions, to familial support systems. These factors, individually or collectively, can either mitigate or exacerbate gender-specific externalizing behaviors in adolescents.

### 4.5. Theoretical contribution and practical implications

The findings derived from this investigation hold significance and offer contributions from dual standpoints, namely theoretical and practical. In terms of theoretical contributions, firstly, this study illuminates the fundamental mechanism by which teacher-student relationship influences the manifestation of externalizing problem behaviors among adolescents. This serves to expand and enhance the current body of empirical research (Hong and Eamon, 2012; Rudasill et al., 2018; Longobardi et al., 2021; Endedijk et al., 2022). Moreover, this study advances our comprehension of the interplay between peer relationship, mental health, parental knowledge, and externalizing problem behaviors from societal perspectives.

Our findings also have practical implications. To improve adolescent externalizing problem behaviors, teachers should take a multifaceted approach. First and foremost, teachers must recognize the critical role that teacher-student relationship plays in addressing externalizing problem behaviors. Teachers should exemplify respect and care towards each student, offering them tangible emotional support through active listening, understanding, and encouragement. This fosters the establishment of a positive relationship with students. Second, teachers should strive to create an inclusive classroom environment that fosters positive peer relationships. This involves being aware of students’ negative peer interactions and taking appropriate measures to minimize them. Third, teachers should also prioritize the mental health of their students. Teachers can achieve this by strengthening their mental health education programs and helping students develop the necessary coping skills to maintain good mental health. Finally, it is imperative for teachers to collaborate with parents and establish a cohesive home-school partnership to support the development of adolescents. Such cooperation can facilitate a comprehensive understanding of the educational and socioemotional needs of students, which can then be addressed with a more holistic and coordinated approach.

## 5. Limitations

This study also has several limitations. First, by only collecting data from three province (Liaoning, Henan and Sichuan Province), the findings may not be generalizable to the entire population. It’s important to obtain a nationally representative sample in the future study to ensure that the findings can be applied to a larger population and to increase the external validity of the study. This would help to guarantee that the results are not specific to the province where the sample was collected and can be applied to the country as a whole. A nationally representative sample would also help to account for any potential regional variations in teacher-student relationship and externalizing problem behaviors. Second, cross-sectional data only provide a snapshot of the relationship between teacher-student relationship and adolescent externalizing problem behaviors at a specific point in time. It’s possible that the relationship between the two variables may change over time, which would not be captured in a cross-sectional study. Longitudinal research in the future study, which follows a group of participants over an extended period of time, would be more informative in understanding the causal relationship between adolescent externalizing problem behaviors and correlates. Third, self-report surveys rely on the participants’ own perspectives, which may not always align with other perspectives. Future studies involving multiple informants, such as parents, peers and teachers, may provide a more comprehensive and rounded understanding of the relationship between teacher-student relationship and externalizing problem behaviors in adolescents. This would also help in identifying any potential biases or inaccuracies in the participants’ self-reported data. Fourth, this study may lack of measurement of other contributors to externalizing problem behaviors. Future research should take other factors into account. Specifically, dimensions such as cultural and ethnic nuances and neurobiological constituents warrant meticulous consideration to attain a comprehensive understanding of the underlying dynamics.

## 6. Conclusion

The present research investigated the manifestations of externalizing problem behaviors among rural adolescents while exploring various preceding factors that exert influence on such behaviors. Specifically, this study unveiled the potential protective effect of teacher-student relationship in mitigating externalizing problem behaviors among rural adolescents. Furthermore, peer relationship and mental health was identified as a potential mediator in the relationship between teacher-student relationship and externalizing problem behaviors among rural adolescents. In addition, this study examined the following mediating pathway: Teacher-student relationship → Peer relationship → Mental health → Externalizing problem behaviors. Moreover, parental knowledge was found to potentially moderate the association between teacher-student relationship and the manifestation of externalizing problem behaviors in rural adolescents. As the level of parental knowledge increases among rural adolescents, the impact of the teacher-student relationship on externalizing problem behaviors becomes more pronounced. Finally, this study found that the impact of teacher-student relationship on rural adolescent externalizing problem behaviors had no significant gender-related differences. This study contributes both theoretically and practically. Firstly, it enhances the existing theoretical framework concerning the connection between teacher-student relationship and externalizing problem behaviors among rural adolescents, shedding light on the underlying mechanisms by which teacher-student relationship impacts the occurrence of such behaviors. Secondly, this study integrates social and individual perspectives in the exploration of potential factors that can ameliorate externalizing problem behaviors among rural adolescents, thus providing a more comprehensive depiction of rural adolescent development. Lastly, the findings of this study offer practical implications for teachers seeking to address and ameliorate adolescent externalizing problem behaviors.

## Data availability statement

The raw data supporting the conclusions of this article will be made available by the authors, without undue reservation.

## Ethics statement

The studies involving humans were approved by the Ethics Institutional Review Board of School of Education at Liaoning Normal University. The studies were conducted in accordance with the local legislation and institutional requirements. Written informed consent for participation in this study was provided by the participants’ legal guardians/next of kin. Written informed consent was obtained from the individual(s), and minor(s)’ legal guardian/next of kin, for the publication of any potentially identifiable images or data included in this article.

## Author contributions

SY: Writing – review & editing, Funding acquisition, Resources, Supervision, Validation. XZ: Writing – original draft, Writing – review & editing, Conceptualization, Formal analysis, Methodology, Software. WL: Writing – original draft, Writing – review & editing. HZ: Writing – review & editing, Writing – original draft.
